# Security evaluation of quantum key distribution with weak basis-choice flaws

**DOI:** 10.1038/s41598-020-75159-6

**Published:** 2020-10-23

**Authors:** Shi-Hai Sun, Zhi-Yu Tian, Mei-Sheng Zhao, Yan Ma

**Affiliations:** 1 School of Physics and Astronomy, Sun Yat-Sen University, Zhuhai, 519082 Guangdong People’s Republic of China; 2 QuantumCTek Co. Ltd., Hefei, 230000 Anhui People’s Republic of China

**Keywords:** Quantum information, Quantum optics

## Abstract

Quantum key distribution (QKD) can share an unconditional secure key between two remote parties, but the deviation between theory and practice will break the security of the generated key. In this paper, we evaluate the security of QKD with weak basis-choice flaws, in which the random bits used by Alice and Bob are weakly controlled by Eve. Based on the definition of Li et al. (Sci Rep 5:16200, 2015) and GLLP’s analysis, we obtain a tight and analytical bound to estimate the phase error and key rate for both the single photon source and the weak coherent source. Our approach largely increases the key rate from that of the original approach. Finally, we investigate and confirm the security of BB84-QKD with a practical commercial devices.

## Introduction

Based on the principle of quantum mechanics, “*quantum cryptography*” is a possible means of implementing unconditional secure communication. One famous quantum cryptography approach is quantum key distribution (QKD) combined with One-Time pad. Since the proposal of the first QKD protocol BB84^1^, QKD has attracted much interest. The unconditional security of QKD had been proven in both perfect^2^ and imperfect^3,4^ devices. QKD has also been experimentally demonstrated in fibers^5,6^, free space^7,8^, and satellites^9,10^. Multi-user quantum networks based on these results have become available in many countries^11–14^.

However, because practical devices are imperfect, some assumptions of the theoretical analysis may be violated in practical situations. If the gap between theory and practice is exploited by an eavesdropper (Eve), the security of the final key may be broken. In fact, many loopholes have been discovered in practical QKD systems^15–21^. These loopholes are closed by two main approaches: device-independent QKD protocols and security patches. The former include full-device-independent QKD^22,23^, measurement-device-independent QKD^24–26^, and semi-device-independent QKD^27^. Security patches account for the parameters of practical devices (as many as possible) in the security model. Although device-independent QKD can remove *all or a portion* of the loopholes, the task remains technologically challenging, especially in practical commercial QKD networks. Thus, most practical QKD systems implement security patches.

In the BB84 protocol, both Alice and Bob must determine how to prepare and measure the quantum states. For this purpose, they require random bits. In practical situations, the random bits may be weakly known or controlled by Eve, and the security of the generated key is compromised. A typical attack that exploits the weak randomness of QKD is wavelength attack^16,17^. The security of QKD with weak randomness was first studied by Li et al.^28^, and has since been applied to different cases^29,30^. In Li’s analysis, if the legitimate parties use the “one-step post processing method” to distill the final key, even a small degree of non-randomness will rapidly reduce the final key rate. The key rate can be improved if the legitimate parties adopt the “two-step post processing method”, or biased basis protocol, in which Alice and Bob distill the key from the rectilinear and diagonal bases, respectively. However, to maximize Eve’s information, they must perform global optimization, which is hampered by at least two disadvantages: large time cost and convergence to a local optimum. The time cost is incurred by the complexity or cost of post processing, and local (rather than global) optimization compromises the security of the generated key.

To mitigate these problems, we develop an analytical formula that estimates the key rate for both the single photon source (SPS) and the weak coherent source (WPS). In numerical simulations, our method significantly increased the key rate over the original method of Li et al.^28^. For example, the original method of Ref.^28^ can generate no secure key for a SPS with a basis-choice flaw of 0.1 when the bit error rate exceeds 3.4%, but our method achieves a final key rate of 0.45 under these conditions. Furthermore, to evaluate the performance of QKD under wavelength attack, we also estimate the key rate of a practical QKD system with a passive basis-choice.

## Results

### Weak randomness and one-step post processing

This section briefly reviews the analysis of Ref.^28^. Alice determines her quantum state from two random bits: $x_0$ for bit and $x_1$ for basis. Meanwhile, Bob chooses his basis from a random bit, *y*. As the final key is distilled only when Alice and Bob choose the same basis ($x_1=y$), the following analysis is limited to the case $x_1=y$. In a practical QKD system, $x_0$ ($x_1$) may be weakly controlled by Eve with a hidden variable $\lambda _0$ ($\lambda _1$). Setting *k* and $k'$ = [0, 1] as the values of $x_0$ and $x_1$, respectively, the probabilities of obtaining $x_0=k$ and $x_1=k'$ are respectively given by

1 $$\begin{aligned} \begin{aligned} p\left( x_{0}=k\right)&=\sum _{i} p_{\lambda _{0}=i} p\left( x_{0}=k | \lambda _{0}=i\right) , \\p\left( x_{1}=k'\right)&=\sum _{j} p_{\lambda _{1}=j} p\left( x_{1}=k' | \lambda _{1}=j\right) . \end{aligned} \end{aligned}$$

Here $\sum _{i} p_{\lambda _{0}=i}=\sum _{j} p_{\lambda _{1}=j}=1$. Due to the existence of the hidden variable $\lambda _0$, we cannot guarantee that $p(x_0=0|\lambda _{0}=i)=p(x_0=1|\lambda _{0}=i)=1/2$ holds for all *i*, even if $p(x_0=0) = p(x_0 =1)=1/2$ holds. The same conclusion is reached for $p(x_1=k')$. To evaluate the weak randomness of $x_0$ and $x_1$, the deviations is defined as

2 $$\begin{aligned} \begin{aligned} \left| p\left( x_{0}=k | \lambda _{0}=i\right) -\frac{1}{2}\right| \le \varepsilon _{0}, \\ \left| p\left( x_{1} =k' | \lambda _{1}=j\right) -\frac{1}{2} \right| \le \varepsilon _{1}, \end{aligned} \end{aligned}$$

respectively. Here, $0 \le \varepsilon _0, \varepsilon _1 \le 1$ define the amount of prior information known to Eve.

When the hidden variables $\lambda _{0}=i$ and $\lambda _{1}=j$ are given, the quantum state shared by Alice and Bob can be written as^28^

3 $$\begin{aligned} \begin{aligned} \rho _{A B}^{i,j}=&\sum _{\mu , \nu } q_{\mu , \nu }\left\{ p\left( x_{1}=0 | \lambda _{1}=j\right) I \otimes X^{\mu } Z^{\nu }|\varphi \rangle \left\langle \left. \varphi \right| _{\lambda _{0}=i} Z^{\nu } X^{\mu } \otimes I\right. \right. \\&+p\left( x_{1}=1 | \lambda _{1}=j\right) I \otimes H X^{\mu } Z^{\nu } H|\varphi \rangle \left\langle \left. \varphi \right| _{\lambda _{0}=i} H Z^{\nu } X^{\mu } H \otimes I\right\} , \end{aligned} \end{aligned}$$

with

4 $$\begin{aligned} |\varphi \rangle _{\lambda _{0}=i}=\sqrt{p\left( x_{0}=0 | \lambda _{0}=i\right) }|00\rangle +\sqrt{p\left( x_{0}=1 | \lambda _{0}=i\right) }|11\rangle . \end{aligned}$$

Here, $\mu ,\nu \in \{0,1\}$, and $q_{\mu ,\nu }$ is the probability that Eve performs different operators on the quantum state $|\varphi \rangle _{\lambda _{0}=i}$. These probabilities satisfy $\sum _{\mu ,\nu } q_{\mu ,\nu }=1$. *I* is the unity matrix, *X* and *Z* are Pauli matrices, and $$H=\frac{1}{\sqrt{2}}\begin{bmatrix}1 &{}1\\ 1 &{} -1\end{bmatrix}.$$

In the following, we first discuss the case of SPS, then expand our results to WPS. In SPS, the total key rate is given by

5 $$\begin{aligned} R \ge \sum _{i,j} p_{\lambda _0=i} p_{\lambda _{1}=j} R^{i,j}. \end{aligned}$$

The key rate for a given *i*, *j* is

6 $$\begin{aligned} R^{i,j}_{sps} \ge 1-H(e_{phase}^{i,j})- H(e_{bit}^{i,j}), \end{aligned}$$

where $e_{bit}^{i,j}$ ($e_{phase}^{i,j}$) is the bit error (phase error) of the given *i*, *j*. Because an experiment reveals only the total bit error $e_{bit} = \sum _{i,j}p_{\lambda _0 =i}p_{\lambda _1 =j} e_{bit}^{i,j}$, Eq. (5) can be rewritten as

7 $$\begin{aligned} \begin{aligned} R_{sps}&= \sum _{i,j} p_{\lambda _0=i} p_{\lambda _{1}=j} \left[ 1-H(e_{phase}^{i,j})- H(e_{bit}^{i,j})\right] \\&\ge 1 -H(\sum _{i,j}p_{\lambda _0=i} p_{\lambda _{1}=j} e_{phase}^{i,j}) -H(e_{bit})\\&\equiv 1-H(e_{phase})- H(e_{bit}). \end{aligned} \end{aligned}$$

Here $e_{phase}$ is the total phase error, and the second inequality uses the Jensen inequality because *H*(*x*) is concave. Before obtaining the lower bound of the key rate, we should estimate the upper bound of $e_{phase}$. The authors of^28^ proved that for the density matrix given by Eq. (3), the upper bound of the phase error can be written as

8 $$\begin{aligned} \begin{aligned} e_{phase}^{i,j}&\le e_{bit}^{i,j} +\delta , \\ e_{phase}= \sum _{i,j}p_{\lambda _0=i} p_{\lambda _{1}=j} e_{phase}^{i,j}&\le \sum _{i,j}p_{\lambda _0=i} p_{\lambda _{1}=j} e_{bit}^{i,j} +\delta =e_{bit} +\delta , \end{aligned} \end{aligned}$$

where

9 $$\begin{aligned} \delta =\max \left\{ \delta _0 \equiv \frac{1}{2}- \sqrt{\frac{1}{4}-\varepsilon _0^2},\quad \delta _1 \equiv 2\varepsilon _1\right\} . \end{aligned}$$

The final key rate of a SPS (Eq. 7) is then rewritten as

10 $$\begin{aligned} R^o_{sps} \ge 1-H(e_{bit} +\delta ) -H(e_{bit}). \end{aligned}$$

In this expression, the superscript *o* distinguishes the original method from our proposed method, which is introduced later. Most practical QKD systems use a WPS. Following GLLP’s analysis^31^, the key rate of Eq. (5) is then written as

11 $$\begin{aligned} \begin{aligned} R^{o}_{wps}&\ge \sum _{i,j}p_{\lambda _{0}=i}p_{\lambda _{1}=j} R^{i,j} \\&\ge \sum _{i,j}p_{\lambda _{0}=i}p_{\lambda _{1}=j} \left\{ -Q_s^{i,j} f(E_{s,bit}^{i,j})H(E_{s,bit}^{i,j}) +Q_{s,1}^{i,j} \left[ 1-H(e_{1,bit}^{i,j}+\delta )\right] \right\} \\&\ge -Q_s f(E_s) H(\sum _{i,j}p_{\lambda _{0}=i}p_{\lambda _{1}=j} E_{s,bit}^{i,j}) +Q_{s,j} \left[ 1 -H(\sum _{i,j}p_{\lambda _{0}=i}p_{\lambda _{1}=j} e_{1,bit}^{i,j}+\delta )\right] \\&\equiv -Q_s f(E_{s,bit}) H(E_{s,bit}) +Q_{s,1} [1- H(e_{1,bit}+\delta )]. \end{aligned} \end{aligned}$$

Here the subscript *s* denotes the key generated from the signal state with intensity *s*. $Q_s^{i,j}$ ($E_{s,bit}^{i,j}$) is the total gain (bit error) for a given *i*, *j*, and $Q_{s,1}^{i,j}$ ($e_{1,bit}^{i,j}$) is the yield (bit error) of the single photon pulse for the given *i*, *j*. The terms $Q_s=\sum _{i,j} p_{\lambda _{0}=i}p_{\lambda _{1}=j} Q_{s}^{i,j}$ ($Q_{s,1}=\sum _{i,j} p_{\lambda _{0}=i}p_{\lambda _{1}=j} Q_{s,1}^{i,j}$) and $E_{s,bit}=\sum _{i,j} p_{\lambda _{0}=i}p_{\lambda _{1}=j} E_{s,bit}^{i,j}$ ($e_{1,bit}=\sum _{i,j} p_{\lambda _{0}=i}p_{\lambda _{1}=j} e_{1,bit}^{i,j}$) are the total gain and error, respectively, for all *i*, *j*. $f(E_{s,bit})=f(E_{s,bit}^{i,j})=1.22$ is the efficiency of the error correction, which can be considered constant. In the third inequality, we recognize that $Q_s \ge Q_{s}^{i,j} \ge 0$ and $Q_{s,1} \ge Q_{s,1}^{i,j} \ge 0$ for all *i*, *j*. The gain $Q_s$ and error $E_{s,bit}$ in the equality can be directly measured in experiments, and the contributions of the single photon pulse ($Q_{s,1}$ and $e_{1,bit}$) should be estimated by the decoy state method^32–34^.

### Our method with one-step post processing

In this section, we show that the upper bound of the phase error (Eq. 8) is suboptimal, and that the key rate can be improved by imposing a tight bound. Given the density matrix $\rho _{AB}^{i,j}$ (Eq. 3), the bit error rate and phase error rate are respectively written as

12 $$\begin{aligned} \begin{aligned} e_{bit}^{i,j}&=\langle \phi _2 |\rho _{AB}^{i,j}|\phi _2 \rangle +\langle \phi _4 |\rho _{AB}^{i,j}|\phi _4 \rangle =q_{01} p(x_1=1|\lambda _1=j) +q_{10} p(x_1=0|\lambda _1=j) +q_{11}\\&\ge \left( \frac{1}{2} -\varepsilon _1 \right) (q_{01}+q_{10}) +q_{11} \equiv e_{bit,low}^{i,j}, \\ e_{phase}^{i,j}&=\langle \phi _3 |\rho _{AB}^{i,j}|\phi _3 \rangle +\langle \phi _4 |\rho _{AB}^{i,j}|\phi _4 \rangle \\&= q_{00} \delta _0 +q_{11} (1-\delta _0)+ q_{01} \left\{ \frac{1}{2}+ \left[ p(x_1=0|\lambda _1=j)- p(x_1=1|\lambda _1=j) \right] (\frac{1}{2}-\delta _0) \right\} \\&\quad +q_{10} \left\{ \frac{1}{2} +[p(x_1=1|\lambda _1=j) -p(x_1=0|\lambda _1=j) ](\frac{1}{2}-\delta _0) \right\} . \end{aligned} \end{aligned}$$

Here, $|\phi _1 \rangle =(|00\rangle +|11\rangle )/\sqrt{2}$, $|\phi _2 \rangle =(|01\rangle +|10\rangle )/\sqrt{2}$,$|\phi _3 \rangle =(|00\rangle - |11\rangle )/\sqrt{2}$,$|\phi _4 \rangle =(|01\rangle - |10\rangle )/\sqrt{2}$ are the four Bell states. Thus we have

13 $$\begin{aligned} \begin{aligned} e_{phase}^{ij}-e_{bit}^{i,j}&= q_{00} \delta _0 -q_{11}\delta _0 +(q_{01}-q_{10}) (1- \delta _0)[2p(x_1=0|\lambda _1 =j) -1]\\&\le q_{00} \delta _0 +q_{11} \delta _0 + 2\varepsilon _1 q_{01} +2\varepsilon _1 q_{10} \\ {}&= (q_{00} +q_{11})\delta _0 +2\varepsilon _1 \frac{e_{bit,low}^{i,j} -q_{11}}{1/2 -\varepsilon _1}\\&=\frac{4\varepsilon _1}{1-2\varepsilon _1} e_{bit,low}^{i,j} -q_{11} \frac{4\varepsilon _1}{1-2\varepsilon _1} +(q_{00} +q_{11})\delta _0 \\&\le \frac{4\varepsilon _1}{1-2\varepsilon _1} e_{bit}^{i,j} +\delta _0, \end{aligned} \end{aligned}$$

where $\delta _0$ is defined in Eq. (9), and $0 \le q_{\mu \nu } \le 1$ for all $q_{\mu \nu }$. Thus, the upper bound of the phase error can be written as

14 $$\begin{aligned} e_{phase}^{i,j} \le \frac{1+ 2\varepsilon _1}{1-2\varepsilon _1} e_{bit}^{i,j} +\frac{1}{2} -\sqrt{\frac{1}{4} -\varepsilon _0^2}. \end{aligned}$$

Submitting the above inequality into Eq. (5) and applying the method described in “Weak randomness and one-step post processing”, the final key rate is rewritten as

15 $$\begin{aligned} R^{t} \ge {\left\{ \begin{array}{ll} 1 - H\left( \frac{1+2\varepsilon _1}{1-2\varepsilon _1} e_{bit} + \delta _0\right) -H(e_{bit}) &{} \text {for SPS}\\ -Q_s f(E_{s,bit}) H(E_{s,bit}) +Q_{s,1} \left[ 1- H\left( \frac{1+2\varepsilon _1}{1-2\varepsilon _1} e_{1,bit} +\delta _0 \right) \right] &{} \text {for WPS} \end{array}\right. }. \end{aligned}$$

**Figure 1:**
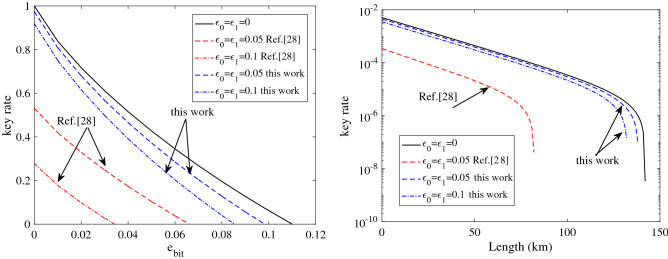
Key rates in the original analysis^28^ (red lines) and the method proposed in this paper (blue lines). Results are plotted for SPS (left) and WPS (right). The black solid line is the result of the ideal case without basis-choice flaws. To simplify the simulation, we assume $\varepsilon _0 =\varepsilon _1$ and infinite decoy states. The WPS case employs the experimental results of GYS^31^; thus, the signal state intensity is $s=0.48$ and the other parameters are set as follows: dark count rate $Y_0 =1.7 \times 10^{-6}$, background error rate $e_0=0.5$, fiber loss 0.21 dB/km, Bob’s transmittance $\eta _{Bob}=0.045$, and error rate of optical devices $e_{det}=3.3\%$. The method of Ref.^28^ generates no key in the case of WPS with $\varepsilon _0 =\varepsilon _1 =0.1$.

Figure 1 compares the numerical simulation results of the method in Ref.^28^ (Eqs. 10, 11) and our method (Eq. 15). Our method significantly improved the key rate for both SPS and WPS (the simulation method is given in “ Formulations of simulation”. For example, in the SPS case with $\varepsilon _0=\varepsilon _1=0.1$, the maximal tolerable error rate was only 3.4% in the method of Ref.^28^, but was increased to 8.5% by our method. In the WPS case with $\varepsilon _0=\varepsilon _1=0.1$, no secure key was generated by the method in Ref.^28^, but a final key of fiber length 132 km was generated by our method.

To evaluate the security of practical QKD with weak randomness flaws, we test the performance of commercial BS which may suffer from the wavelength attack. The experimental scheme and results are given in “Formulations of simulation”, and the estimated key rate is listed in Table 1.

**Table 1:** Key rates estimated by our method.

T	$-5^\circ$	$18^\circ$	$25^\circ$	$40^\circ$	$70^\circ$
$\varepsilon _1$	0.0253	0.0235	0.0250	0.0274	0.0275
$\gamma$ (SPS)$@ e_{bit}=3\%$	0.0259	0.0239	0.0255	0.0281	0.0282
$\gamma$ (SPS)$@ e_{bit}=5\%$	0.0521	0.0482	0.0514	0.0566	0.0567
$\gamma$ (SPS)$@ e_{bit}=7\%$	0.1017	0.0941	0.1004	0.1105	0.1108
$\gamma$ (WPS)$@ L=10\,\hbox { km}$	0.0445	0.0411	0.0438	0.0483	0.0484
$\gamma$ (WPS)$@ L=50\,\hbox { km}$	0.0454	0.0420	0.0448	0.0493	0.0495
$\gamma$ (WPS)$@ L=100\, \hbox { km}$	0.0548	0.0507	0.0541	0.0596	0.0597

Here $\gamma =(R_{ideal}-R_{prac})/R_{ideal}$defines the practical key rate ($R_{prac}$) relative to the ideal key rate without basis-choice flaws ($R_{ideal}$). In the simulations, we set $\varepsilon _0 =0$, and the other parameters were those assumed in Fig. 1.

### Biased base QKD protocol

In some practical QKD systems, two bases (*Z* and *X*) can deliver different gain or error rate performances. Therefore, to improve the total key rate, we let Alice and Bob observe bases *Z* and *X*, respectively. In this section, we analyze the security of biased base QKD with weak randomness. When Alice and Bob distill the key from their respective bases, the key rate becomes

16 $$\begin{aligned} R^{two}_{SPS} \ge p_{rec}[1-H(e_{rec}^b) -H(e_{rec}^p)] +p_{dia} [1- H(e_{dia}^b) -H(e_{dia}^p)]. \end{aligned}$$

Here $e_{rec}^b$ and $e_{rec}^p$ ($e_{dia}^b$ and $e_{dia}^p$) are the bit error and phase error rates, respectively, in the rectilinear (diagonal) basis. Their values are given by

17 $$\begin{aligned} \begin{aligned} e_{rec}^p&= \frac{p_{rec1} e_{b 01}+p_{rec2} e_{b 11}}{p_{rec}} +\frac{1}{2}-\sqrt{-\epsilon _{0}^{2}+\frac{1}{4}}, \\ e_{dia}^p&= \frac{p_{\text{ dial }} e_{b 00}+p_{\text{ dia } 2} e_{b 10}}{p_{\text {dia}}}+\frac{1}{2}-\sqrt{-\epsilon _{0}^{2}+\frac{1}{4}}\\e_{rec}^b&=\frac{p_{\text{ rec1 }} e_{b 00}+p_{\text{ rec } 2} e_{b 10}}{p_{\text{ rec }}}, \\e_{dia}^b&=\frac{p_{\text{ dial }}, e_{b 01}+p_{\text{ dia } 2} e_{b 11}}{P_{\text{ dia }}}. \end{aligned} \end{aligned}$$

In these expressions, $e_{b00}$ and $e_{b01}$ ($e_{b10}$ and $e_{b11}$) are the bit error rates in the rectilinear and diagonal bases, respectively, given a hidden variable $\lambda _1=0$ ($\lambda _1 =1$). $p_{rec}$ and $p_{dia}$ are the probabilities that Bob obtains the outcome in the rectilinear and diagonal bases, respectively. They are calculated as

18 $$\begin{aligned} p_{rec}=p_{rec1}+ p_{rec2}, \quad p_{dia} =p_{dia1}+ p_{dia2}, \end{aligned}$$

respectively, where $p_{rec1}=p_{\lambda _{1}=0} p\left( x_{1}=0 | \lambda _{1}=0\right)$, $p_{rec2}=p_{\lambda _{1}=1} p\left( x_{1}=0 | \lambda _{1}=1\right)$, $p_{dia1}=p_{\lambda _{1}=0} p\left( x_{1}=1 | \lambda _{1}=0\right)$, and $p_{dia2}=p_{\lambda _1=1} p\left( x_1=1|\lambda _1=1 \right)$.

As the bit error rates $e_{rec}^b$ and $e_{dai}^b$ can be directly measured in experiments, we need only to estimate the upper bounds of the phase error rates $e_{rec}^p$ and $e_{dai}^p$. Using Eq. (17), the phase error $e_{rec}^p$ becomes

19 $$\begin{aligned} \begin{aligned} e_{rec}^p&=e_{dia}^b \frac{p_{dia}}{p_{rec}}\frac{p_{rec1}e_{b01} +p_{rec2}e_{b11}}{p_{dia1}e_{b01} +p_{dia2}e_{b11}} +\delta _0 \\&= e_{dia}^b \frac{p_{dia}}{p_{rec}}\frac{p_{\lambda _1=0} p(x_1=0|\lambda _1=0) e_{b01} +p_{\lambda _1=1} p(x_1=0|\lambda _1=1) e_{b11}}{p_{\lambda _1=0} p(x_1=1|\lambda _1=0) e_{b01} +p_{\lambda _1=1} p(x_1=1|\lambda _1=1)e_{b11}} +\delta _0 \\&\le e_{dia}^b \frac{p_{dia}(1/2 +\varepsilon _1)}{p_{rec}(1/2 -\varepsilon _1)} +\delta _0. \end{aligned} \end{aligned}$$

By the above method, we also obtain

20 $$\begin{aligned} e_{dia}^p \le e_{rec}^b \frac{p_{rec}(1/2 +\varepsilon _1)}{p_{dia}(1/2 -\varepsilon _1)} +\delta _0. \end{aligned}$$

Here we assume that the bit weak randomness in Z-basis is the same as that of X-basis, thus the same $\delta _0$ is used in the equations above. But, by considering $\delta _0$ in the bases respectively, our analysis is also valid for the QKD system with different bit weak randomness.Then Eq. (16) can then be written as

21 $$\begin{aligned} \begin{aligned} R^{two}_{SPS}&\ge p_{rec} \left\{ 1-H(e_{rec}^b) -H\left[ e_{dia}^b \frac{p_{dia}(1/2 +\varepsilon _1)}{p_{rec}(1/2 -\varepsilon _1)} +\delta _0\right] \right\} \\&\quad +p_{dia} \left\{ 1-H(e_{dia}^b) -H\left[ e_{rec}^b \frac{p_{rec}(1/2 +\varepsilon _1)}{p_{dia}(1/2 -\varepsilon _1)} +\delta _0\right] \right\} . \end{aligned} \end{aligned}$$

Applying the method above and GLLP’s analysis, the key rate given by Eq. (21) can be expanded to the WPS case as follows:

22 $$\begin{aligned} \begin{aligned} R_{wps}^{two}&\ge p_{rec}\left\{ - Q_{s,rec} f(E_{s,rec}^b) H(E_{s,rec}^b) +Q_{1,rec}\left[ 1 - H(e_{1,dia}^b \frac{p_{dia}(1/2+\varepsilon _{0})}{p_{rec}(1/2-\varepsilon _{1})} +\delta _0)\right] \right\} \\&\quad + p_{dia}\left\{ - Q_{s,dia} f(E_{s,dia}^b) H(E_{s,dia}^b) +Q_{1,dia}\left[ 1 - H(e_{1,rec}^b \frac{p_{rec}(1/2+\varepsilon _{0})}{p_{dia}(1/2-\varepsilon _{1})} +\delta _0)\right] \right\} , \end{aligned} \end{aligned}$$

where $Q_{s,rec}$ ($Q_{s,rec}$) and $E_{s,dia}^b$ ($E_{s,dia}^b$) denote the total gain and bit error rate, respectively, in the rectilinear (diagonal) base, and $Q_{1,rec}$ ($Q_{1,rec}$) and $e_{1,dia}^b$ ($e_{1,dia}^b$) are the gain and bit error rate, respectively, of a single photon pulse in the rectilinear (diagonal) base.

## Discussion

We evaluated the security of QKD with weak basis-choice flaws. The previous analysis of Li et al.^28^ was extended by applying a tight analytical bound for estimating the phase error. The final key rate was significantly improved by the proposed approach. For example, when $\varepsilon _0=\varepsilon _1=0.1$ and the bit error rate exceeded 3.4%, no final key was generated by the previous method, but a final key rate of 0.45 was achieved by our method. Applying our analysis, we evaluated the security of a practical QKD system in which Bob passively chooses his basis with a BS. In experiments using a practical BS with typical parameters, the key rate was reduced by less than 6%. Thus, the proposed method improves the QKD performance even in weak randomness scenarios.

Note that, although we analyze the weak randomness of basis-choice in this paper, there are other imperfections in source and detection. Thus, how to take all of these imperfections in one general mode is still an open question, and we will discuss it in our further works.

## Methods

### Formulations of simulation

This Appendix shows the simulation formulations of Fig. 1 and Table 1. In the absence of Eve, the total gain and error rate are respectively written as

23 $$\begin{aligned} \begin{aligned} Q_\omega&= 1-(1-Y_0)e^{-\omega \eta }\\ and\; E_\omega Q_\omega&=e_0 Y_0 +e_{det}(1-e^{-\omega \eta }). \end{aligned} \end{aligned}$$

Here, $\omega \in \{s\}$ and $\omega \in \{d\}$ are the intensities of the signal state (*s*) and decoy state (*d*), respectively, $Y_0$ is the dark count of the single photon detector, and $e_0$ is the background error rate. $\eta$ is the total transmittance of the system, which is given by

24 $$\begin{aligned} \eta =\eta _{Bob} 10^{-\alpha /10}. \end{aligned}$$

In this expression, $\eta _{Bob}$ is the transmittance of Bob’s optical devices and the efficiency of single-photon detectors, and $\alpha$ is the channel loss. In QKD with a weak coherent source, photon-number-dependent attacks (such as photon-number splitting attacks) must be removed by the decoy state method. Assuming that Alice and Bob use infinite decoy states, the gain and quantum bit error rate of a single photon pulse are respectively given by

25 $$\begin{aligned} \begin{aligned} Q_{s,1}&= s e^{-s} [1- (1-Y_0)(1-\eta )] \equiv s e^{-s} Y_1,\\ e_1&=(e_0 Y_0 +e_{det} \eta )/ Y_1. \end{aligned} \end{aligned}$$

### Experiment

**Figure 2:**
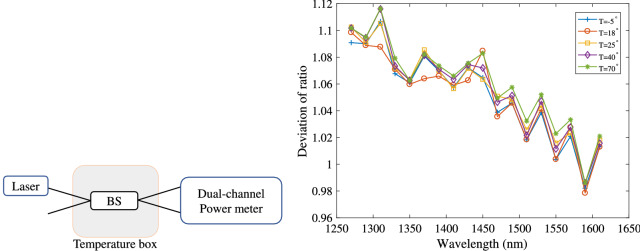
Experimental scheme (left) and measured deviation ratio (right) of a practical commercial BS that evaluates the key rate under a weak measured basis flaw. The deviation ratio is determined by Eq. (26) and *T* is the working temperature of the BS, which is controlled by the temperature box.

In some practical QKD systems, Bob passively chooses his measured basis with a BS. Because this scheme requires no active modulator, it enables high-speed, low-cost, and low-complexity operations. However, (as is well known) the transmittance of the BS may depend on the wavelength of the light, opening a potential loophole for wavelength attack by Eve^16,17^. In this section, we evaluate the performance of QKD with a passive BS by the above analysis.

The experimental scheme is shown in the left panel of Fig. 2. The BS was encased in a temperature box that controlled its working temperature. The input of the BS was a tunable laser (model JW3113; province, country), and the light output was measured by a dual-channel power meter (model JW8103D, province, country). In a perfect BS, the measured power of both power meters is identical. The performance of a real BS is defined by its deviation ratio as follows:

26 $$\begin{aligned} \Delta (T,\lambda ) = \frac{P_0(T,\lambda )}{P_1(T,\lambda )}. \end{aligned}$$

Here, $P_0$ and $P_1$ are the powers of the light measured by the two optical power meters, *T* is the working temperature of the BS, and $\lambda$ is the wavelength of the input light.

The measured deviation ratio of the BS was measured at different wavelengths of the input light and different working temperatures. The results are shown in the right panel of Fig. 2. From the experimental results, the weak randomness in Bob’s basis-choice can be estimated as

27 $$\begin{aligned} \varepsilon _1 =\max _{\lambda } \left| \frac{P_0}{P_0 +P_1} -\frac{1}{2} \right| = \max _{\lambda } \left| \frac{\Delta }{1+ \Delta } -\frac{1}{2} \right| . \end{aligned}$$

The estimated key rates in the SPS and WPS cases are listed in Table 1 (see main text). At communication distances smaller than 100 km, the key rate was reduced by less than 6%. Topical subheadings are allowed. Authors must ensure that their “Methods” section includes adequate experimental and characterization data necessary for others in the field to reproduce their work.
